# A comparative study of the microbiomes of the ticks *Rhipicephalus microplus* and *Hyalomma anatolicum*[Fn FN1]

**DOI:** 10.1051/parasite/2024074

**Published:** 2024-11-28

**Authors:** Adeel Mumtaz Abbasi, Shiza Nasir, Amna Arshad Bajwa, Haroon Akbar, Muhammad Muddassir Ali, Muhammad Imran Rashid

**Affiliations:** 1 Department of Parasitology, University of Veterinary & Animal Sciences 54000 Lahore Pakistan; 2 Institute of Biochemistry and Biotechnology, University of Veterinary & Animal Sciences 54000 Lahore Pakistan

**Keywords:** Tick microbiome, V3-V4 16S rRNA sequencing, *Rhipicephalus microplus*, *Hyalomma anatolicum*

## Abstract

*Hyalomma anatolicum* and *Rhipicephalus microplus* are tick species that are important vectors of numerous pathogens affecting both humans and livestock. Endosymbionts, such as *Coxiella*-like endosymbionts (CLE), *Francisella*-like endosymbionts (FLE)*,* and *Candidatus* Midichloria, play a crucial role in the physiology and vector competence of these ticks. In this study, we investigated the microbial composition of *H. anatolicum* and *R. microplus* from four geographically distinct regions of Pakistan to assess whether environmental differences influence their microbiomes. We analyzed the ticks’ gut microbiome targeting the V3-V4 hypervariable region of 16S rRNA for Illumina 16S metagenome NGS sequencing and processed overall 144 ticks. Analysis of gut bacterial composition resulted in observation of 1200 *R. microplus* and 968 *H. anatolicum* unique amplicon sequencing variants (ASVs). Relative abundance, Alpha diversity (Shannon, Faith’s phylogenetic distance) and beta diversity metrics (Bray–Curtis, Jaccard and UniFrac) were analyzed and revealed that *H. anatolicum* ticks have significantly unique and diverse microbial communities with *Acinetobacter indicus* and *Francisella*-like endosymbionts dominating as opposed to *Candidatus* Midichloria. *Rhipicephalus microplus* exhibited results consistent with the previous studies with no major changes in microbiome including *Coxiella*-like endosymbionts as the major contributor. These findings suggest that geographical and environmental factors play a significant role in shaping the tick microbiome, with potential consequences for disease transmission and tick survivability. Further research is needed to elucidate the functional roles of these microbial shifts and their impact on public health and livestock in affected regions.

## Abstract (fr)

*Hyalomma anatolicum* et *Rhipicephalus microplus* sont des espèces de tiques qui sont d’importants vecteurs de nombreux agents pathogènes affectant à la fois les humains et le bétail. Les endosymbiontes, tels ceux de type *Coxiella* (CLE), de type *Francisella* (FLE) et *Candidatus* Midichloria, jouent un rôle crucial dans la physiologie et la compétence vectorielle de ces tiques. Dans cette étude, nous avons étudié la composition microbienne de *H. anatolicum* et *R. microplus* de quatre régions géographiquement distinctes du Pakistan pour évaluer si les différences environnementales influencent leurs microbiomes. Nous avons analysé le microbiome intestinal des tiques en ciblant la région hypervariable v3-v4 de l’ARNr 16S pour le séquençage 16S NGS Illumina du métagénome et avons traité au total 144 tiques. L’analyse de la composition bactérienne intestinale a permis d’observer 1200 (*R. microplus*) et 968 (*H. anatolicum*) variants de séquençage d’amplicons (ASV) uniques. L’abondance relative, la diversité alpha (distance phylogénétique de Shannon, Faith) et les mesures de diversité bêta (Bray–Curtis, Jaccard et UniFrac) ont été analysées, ce qui a révélé que les tiques *H. anatolicum* ont des communautés microbiennes particulièrement uniques et diversifiées, avec des endosymbiontes de type *Acinetobacter indicus* et *Francisella* dominants, par opposition à *Candidatus* Midichloria. *Rhipicephalus microplus* a présenté des résultats cohérents avec les études précédentes, sans changement majeur du microbiome, y compris les endosymbiontes de type *Coxiella* comme contributeurs majeurs. Ces résultats suggèrent que les facteurs géographiques et environnementaux jouent un rôle important dans la formation du microbiome des tiques, avec des conséquences potentielles sur la transmission des maladies et la survie des tiques. Des recherches supplémentaires sont nécessaires pour élucider les rôles fonctionnels de ces changements microbiens et leur impact sur la santé publique et le bétail dans les régions touchées.

## Introduction

Ticks are blood sucking obligate ectoparasites that belong to the family Ixodidae and infest a variety of animals and humans [4]. About 28 species of ticks act as vectors of tick-borne infections to humans [8]. Tick bites can result in diseases like Crimean-Congo Haemorrhagic Fever (CCHF), Lyme disease, tick paralysis, relapsing fever, anaplasmosis, tularemia and spotted fever rickettsiosis in humans. *Hyalomma anatolicum* ticks are known for spreading CCHF infection in humans and cattle [29, 36, 43]. Tick-borne bacterial infections namely *Francisella, Ehrlichia, Borrelia, Rickettsia* and *Anaplasma* to humans are a major public health concern. Infection by these pathogens can have lifelong detrimental effects on host health [22]. These ticks not only transmit viruses, bacteria, and protozoa, but also harbor endosymbionts, intracellular bacteria that live in symbiotic relationships within the ticks themselves.

Tick endosymbionts were first described by Cowdry in the early 20th century. Recent studies have proved that a diverse tick microbiome composition is crucial for its health status and reproductive potential [13, 21, 44, 45]. Endosymbionts like FLE, CLE, and *Candidatus* Midichloria are frequently associated with ticks and have garnered attention for their unique interactions with their tick hosts. While some of these bacteria are non-pathogenic and contribute to the tick’s biology – such as reproduction, metabolism, and survival – others, like pathogenic species of FLE, can also pose a direct threat to human health. CLE bacteria play crucial roles in tick physiology. These symbionts provide essential nutrients, like B vitamins, that ticks cannot acquire from their blood meals alone [16, 20]. Midichloria, on the other hand, represents a fascinating genus known for its association with mitochondria, although its full role in tick biology remains under investigation. It has also been found to be a dual-partner in the nutritional system [9]. *Acinetobacter indicus*, which is prevalent in both *Hyalomma* and *Rhipicephalus* ticks, has also been observed to produce thermostable chitinase extracellularly (Strain CCS-12), which has anti-fungal properties [3]. Endosymbionts can interact with the pathogens that ticks carry, influencing their transmission to hosts. Some symbionts may compete with pathogens for resources or space within the tick, potentially reducing the tick’s ability to transmit diseases like Lyme disease, anaplasmosis, or spotted fever. In contrast, other symbionts might enhance pathogen survival or transmission [20].

Evidence of the impactful relationship between tick-borne pathogens and the microbiome has been recorded in a previous study which states that *Anaplasma phagocytophilum* has the ability to modulate the composition of tick microbiome and its species richness [19]. Ticks use different mechanisms to adapt to these microbes. For example, *Ixodes scapularis* gut infected with *B. burgdorferi* and *A. phagocytophilum* causes changes in gene expression of the tick [1]. Similarly, these tick species when collected from the field with rich and competitive microbiome profiles resist *B. burgdorferi* uptake and survival [6, 14].

Tick endosymbionts have been observed to modulate tick physiology and transmit rickettsial agents from ticks to mammals. *Rickettsia peacockii* changes tick physiology in such a way that it develops a negative correlation for transmission of *R. rickettsii* from *Dermacentor* tick to animals [11]*.* These findings revealed that the relationship between endosymbionts and pathogens within a tick has an impact on tick biology and has become an important topic to understand ticks. A few samples of *H. anatolicum* and *R*. *microplus* ticks from Pakistan have been tested for metagenomic analysis, which has provided insight into the pathogenic infections of these ticks [2]. However, this area of research is largely unexplored for developing a tick metagenome profile of bovine ticks of Pakistan.

Complexity of tick microbiome relationship with its host has recently been explored with greater success and significant findings because of the application advantages of NGS sequencing but this aspect of scientific exploration is neglected in Pakistan. Metagenomic studies provide a picture of potential pathogens carried by these arthropods of livestock and public health importance [49]. Understanding these microbes’ relationships with tick health and their vectorial potential can help to devise strategies to counter ticks and tick-borne diseases in the future [18, 30]. About 78% of the livestock in Pakistan is affected by ticks, and *H. anatolicum* is the most prevalent tick species in arid areas of the country [41]. *Hyalomma anatolicum* and *R. microplus* pose significant economic problems for the livestock production in Pakistan. For example, *R. microplus* transmits *Babesia bovis*, *Anaplasma marginale*, and *Babesia bigemina* in cattle, whereas *Hyalomma* ticks transmit *Theileria annulata* in cattle and CCHF from cattle to humans [37]. In another study, *R. microplus* and *H*. *anatolicum* prevalence was reported throughout Pakistan. In these ticks, *Candidatus* Rickettsia amblyommii was seen in *R. microplus* and *H*. *anatolicum* ticks which cause spotted fever in humans and theileriosis in bovines. *Hyalomma dromedarii* ticks were found to be infected with *T*. *annulata* which cause significant losses in livestock in Pakistan [23].

Studying the symbiotic relationships between ticks and their endosymbionts provides insights into co-evolution and the ecological dynamics of tick populations. The presence of specific symbionts may indicate geographic origin or evolutionary history. This can help researchers track tick migrations, understand the spread of tick-borne diseases, and develop more targeted surveillance and control measures. Moreover, understanding these interactions may reveal vulnerabilities that can be exploited to disrupt tick development and reduce tick populations. For example, symbionts could be targeted with antimicrobials, vaccines, or genetic strategies, which could reduce tick viability or reproductive success.

In this study, comprehensive microbiome diversity profiles in different provinces of Pakistan were evaluated to assess whether there has been any change in the microbial composition of these ticks. The microbiome can affect the vectorial potential of ticks and Pakistan has diverse geographic and climatic domains spanning from cooler mountainous areas to hotter deserts, which may be the reason why one particular province has reported increasing cases of CCHF and theileriosis than the others [24, 47]. Our research highlights significant changes in the *H. anatolicum* microbiome and reported that, at the same time, the *R. microplus* microbiome was consistent with previous reports.

## Materials and methods

### Tick sample processing

*Hyalomma anatolicum* (*n* = 72) and *Rhipicephalus microplus* (*n* = 72) ticks were collected from Punjab (PB), Khyber Pakhtunkhwa (KPK), Balochistan (BN) and Sindh (SH) provinces, Pakistan. Tick sample pre-processing and microscopic identification of tick species were performed according to the method described elsewhere [26, 28, 50]. Eighteen engorged adult female ticks were pooled into three replicates from each province. After tick gut dissection, DNA was extracted using ZymoBIOMICS 96 DNA Kits (ZYMO Research, Irvine, CA, USA) and stored at −20 °C until use.

### 16S rRNA gene library preparation and sequencing

The 16S rRNA gene V3-V4 region was amplified by the PCR primers recommended by Illumina, as follows:

16S-Forward Primer = 5′ TCGTCGGCAGCGTCAGATGTGTATAAGAGACAGCCTACGGGNGGCWGCAG 3′ 16S-Reverse Primer = 5′ GTCTCGTGGGCTCGGAGATGTGTATAAGAGACAGGACTACHVGGGTATCTAATCC 3′. The underlined section of primer bases are overhang adaptor sequences incorporated with the V3-V4 hypervariable region-specific primers [35]. The 16S rRNA V3-V4 amplification was done by NEB Q5^®^ Hot Start High-Fidelity 2X Mix Master Mix (New England Biolabs, Ipswich, MA, USA). PCR conditions were set as 95 °C for 3 min followed by 25 cycles of 95 °C for 30 s, 55 °C for 30 s, 72 °C for 30 s and final extension of 5 min at 72 °C. PCR products were verified on a Bioanalyzer DNA 1000 chip for quality of amplicons. DNA/amplicon purifications were performed by AMPure XP magnetic beads (Agencourt AMPure, Beckman Coulter, Brea, CA, USA). The 16S Illumina Metagenome Library was prepared by following the protocol recommended by Illumina (USA) (https://support.illumina.com/downloads/16s_metagenomic_sequencing_library_preparation.html). An Illumina Nextera^®^ XT Index Kit (FC-131-1001) was used according to protocol (Illumina, San Diego, CA, USA). Further procedures previously described in another study were followed [2]. PCR product with amplified 16S amplicon was purified with magnetic beads. NGS amplicon sequencing libraries were quantified and checked for quality and average fragment size using a Qubit fluorometer (Thermo Fisher Scientific, Waltham, MA, USA) and Bioanalyzer (Agilent 2100, Agilent, Santa Clara, CA, USA), following manufacturers’ protocols. A MiSeq Reagent Kit v3 (MS-102-3003) 600 cycles was used for the sequencing run. The Illumina PhiX Control v3 Library (Illumina) was used for sequencing quality control.

### Bioinformatics and statistical analyses

Demultiplexed sequence files retrieved from the MiSeq instrument were processed on high throughput Computer using Linux based software. Metagenomic analysis methods with a command line interface (CLI) of Quantitative Insights into Microbial Ecology 2 (QIIME 2) Linux Distribution v2023.8 were deployed to analyze all the pooled samples [7]. Analysis steps were also reassessed on the QIIME2 Galaxy Server (Cancer Galaxy Server, https://cancer.usegalaxy.org) to verify the process. The QIIME2 Moving Pictures Guide pipeline was used for most of the analyses (https://docs.qiime2.org/2024.2/tutorials/). Sections of Atacama Soil Microbiome and Parkinson’s Mouse metagenomic pipeline were also incorporated into the analysis, where relevant. Integrity and authenticity of tabular metadata was verified by cloud based Keemei plugin. The Divisive Amplicon Denoising Algorithm (DADA2) plugin was used to denoise, filter and rejoin forward and reverse sequences. Amplicon sequence variants (ASVs) generated via DADA2 were subsequently utilized throughout further analysis [10]. Pre-trained Naive Bayes classifier at 99% similarity (silva-138-99-seqs.qza) was used as for taxonomy assignment [30]. MAFFT and FastTree extensions of QIIME2 were deployed for sequence alignment and phylogenetic tree construction [40]. Alpha and beta diversity were analyzed by *qiime diversity core-metrics-phylogenetic*, *qiime diversity alpha-group-significance* and *qiime diversity beta-group-significance* functions [17]. The ASVs feature table and feature data summaries were obtained using *qiime feature-table summarize* and *qiime feature-table tabulate-seqs* functions. Plots were generated using *qiime taxa barplot*, *qiime emperor plot* and *qiime diversity alpha-rarefaction* plugins. Data visualization including relative abundance profile and alpha and beta diversity visualization was performed with the Qiime2View online web tool by utilizing QIIME proprietary QZV artifacts obtained during the process (https://view.qiime2.org).

### Alpha and beta diversity

The samples were analyzed for the Shannon–Wiener diversity index (Shannon index), Pielou’s measure of species evenness to estimate species richness and evenness across the samples based on observed ASVs, and relative abundance. Faith’s phylogenetic diversity (Faith_PD) index was also performed to account for the phylogenetic differences among observed species. For beta diversity, Bray–Curtis dissimilarity, Jaccard distance and UniFrac Distance (unweighted UniFrac and weighted UniFrac) were performed to evaluate the distance/dissimilarity between all the tick-groups [31]. Principal coordinates analysis (PCoA) was performed for their respective beta diversity metrics to visualize the community differences between the groups.

### Statistical analysis

Samples from different provinces in the context of categorical metadata were analyzed for alpha diversity using the Kruskal–Wallis test to assess the change in richness and evenness within the groups (provinces) and the permutational multivariate ANOVA (PERMANOVA) statistical test was performed to analyze the diversity between groups/provinces (beta diversity).

## Results

### Illumina 16S rRNA (V3–V4) sequencing and bioinformatic analyses

In the case of *R. microplus*, 7.6 million reads were obtained from all 12 pools with an average of 636,000 reads per sample, while *H. anatolicum* pools yielded 6.9 million reads with an average of 575,000 reads per sample. Overall, 93.7% of clusters passed the QC filter with 649 k/mm^2^ cluster density. Raw sequences data were submitted into the NCBI SRA Database under the BioProjects PRJNA1100935 and PRJNA1098064 for *R. microplus* and *H. anatolicum*, respectively. After quality filtering, the forward and reverse reads were aligned and non-chimeric reads were excluded. *R. microplus* and *H. anatolicum* sequences resulted in 1200 and 968 unique ASVs, respectively. Each sample was rarefied at 5000 sequences to facilitate the analysis computation.

### Comparative analysis of bacterial communities

**Figure 1:**
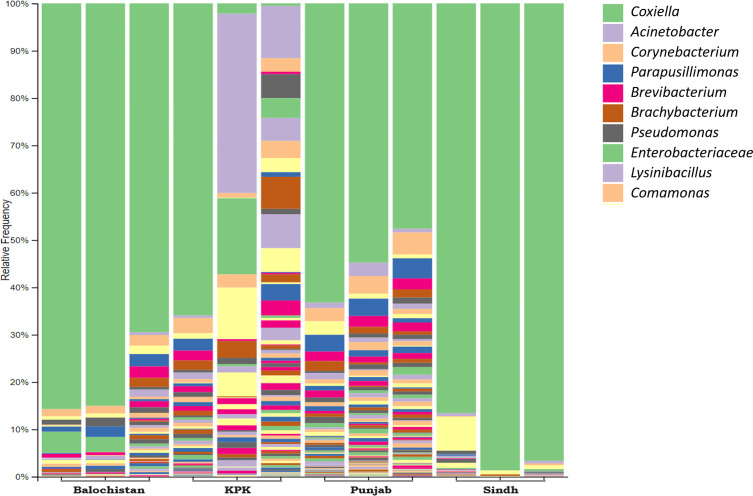
Relative abundance showing that the *Coxiella* and *Acinetobacter* genera are dominant in all the samples. *Coxiella*-like endosymbionts occupied most of the *Rhipicephalus microplus* microbial composition from all the provinces except KPK, where *Acinetobacter indicus* is the dominant bacterium. Legend represents colors coding from top to bottom of abundance bars.

**Figure 2:**
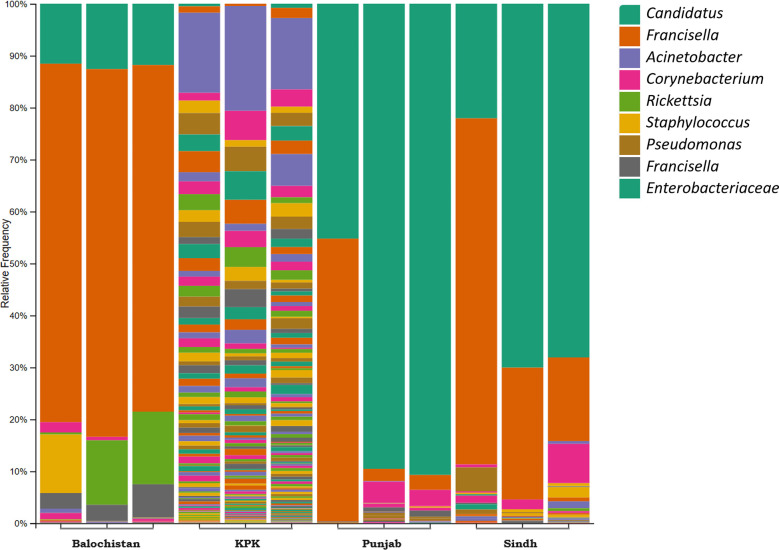
*Hyalomma anatolicum* microbial abundance showing *Candidatus* Midichloria and *Francisella-*like endosymbionts (FLE) as the dominant genera of bacteria in all the samples, but KPK. Legend represents colors coding from top to bottom of abundance bars.

The *Coxiella*-like endosymbionts (CLE) constituted more than half of the *R. microplus* microbial community from pools of PB, SH and BN ticks; CLE were comparatively less abundant in KPK ticks. *Acinetobacter indicus* constituted around 17% of the KPK *R. microplus* microbiome as a major contributor (Fig. 1)*.* In the case of *H. anatolicum,* the most abundant genus was *Candidatus* Midichloria in eastern provinces of PB and SH. However, *A. indicus* and *FLE* were observed in the majority of KPK and BN samples of *H. anatolicum*, respectively (Fig. 2).

### Alpha diversity

**Figure 3:**
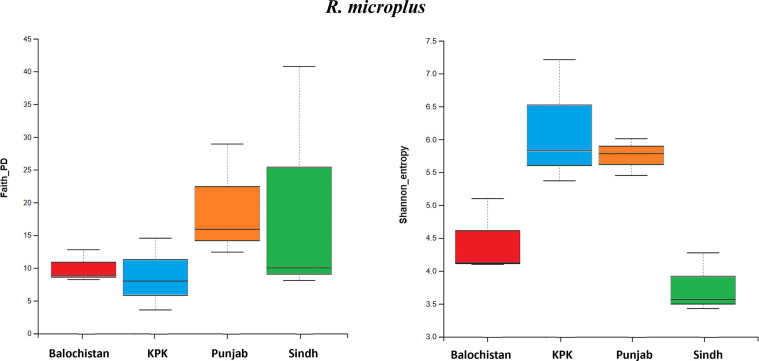
*Rhipicephalus microplus*: Faith’s Phylogenetic Diversity index (Faith_PD) *p*-value = 0.31, H-value = 3.51*.* Shannon Index (Shannon_entropy) *p*-value = 0.03, H-value = 8.64*.*

**Figure 4:**
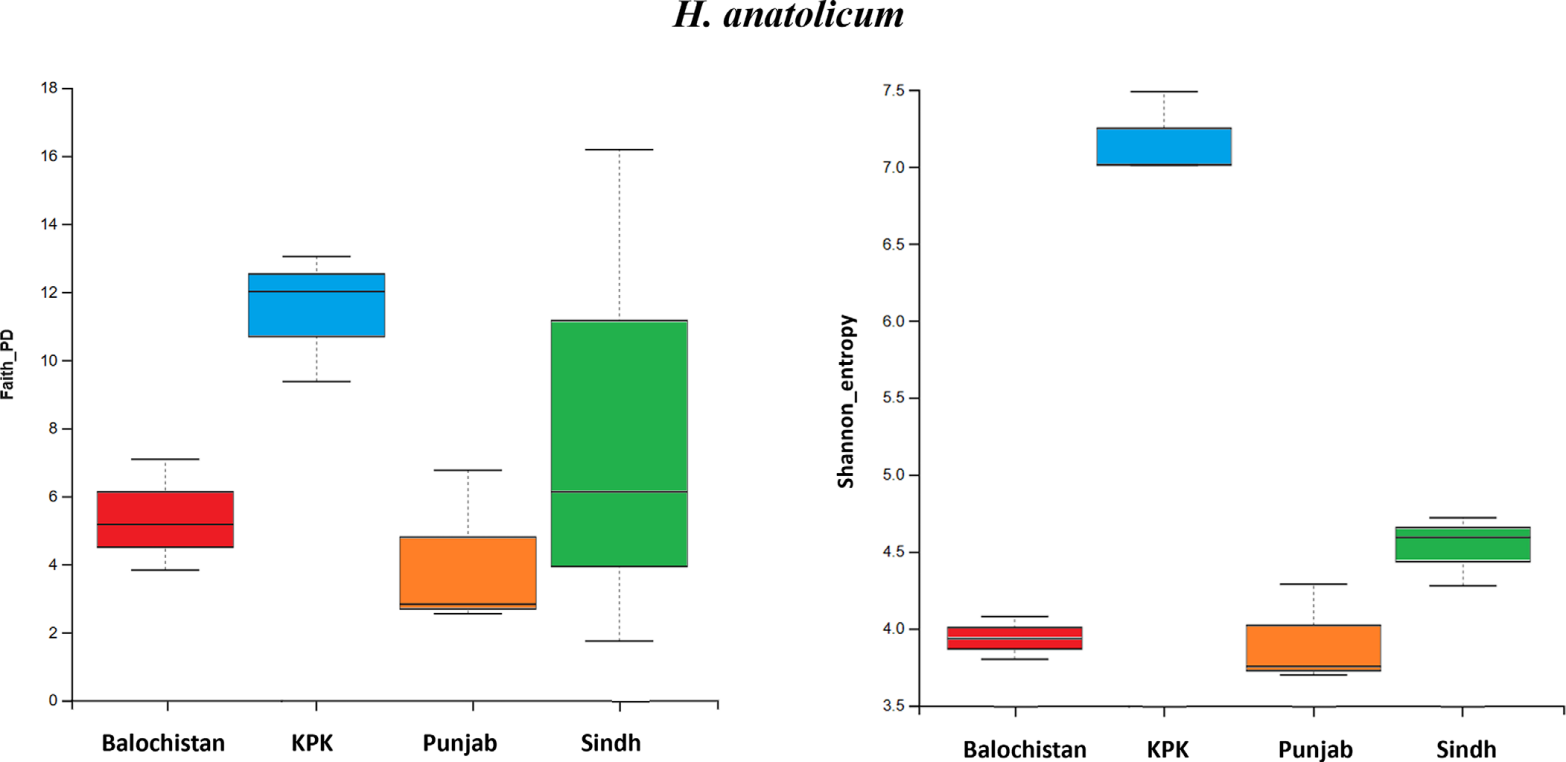
*Hyalomma anatolicum:* Faith’s Phylogenetic Diversity index (Faith_PD), *p*-value = 0.21, H-value = 4.43. Shannon Index (Shannon_entropy), *p*-value = 0.03, H-value = 8.7.

Microbial communities of *R. microplus* ticks were significantly diverse but phylogenetically not unique to each province. Also, the *R. microplus* Faith’s Phylogenetic Diversity index (Faith_PD) test did not show any significant difference within groups (*p*-value = 0.31, H-value = 3.51). The Shannon index showed significant diverse microbial communities within the *R. microplus* tick groups (Shannon index H-value = 8.64 and *p*-value = 0.03, Pielou’s evenness H-value 8.74, *p*-value = 0.03) (Fig. 3). Similarly, *H. anatolicum* did not present any significant diversity and evenness of species distribution within the groups (Faith-PD *p*-value = 0.21, H-value = 4.43; Pielou’s Evenness *p*-value = 0.31, H-value = 5.56) (Fig. 4). Nonetheless, there was enough richness to account for a statistically significant result when compared to the Shannon index (*p*-value = 0.03, H-value = 8.7) (Fig. 4).

### Beta diversity

**Figure 5:**
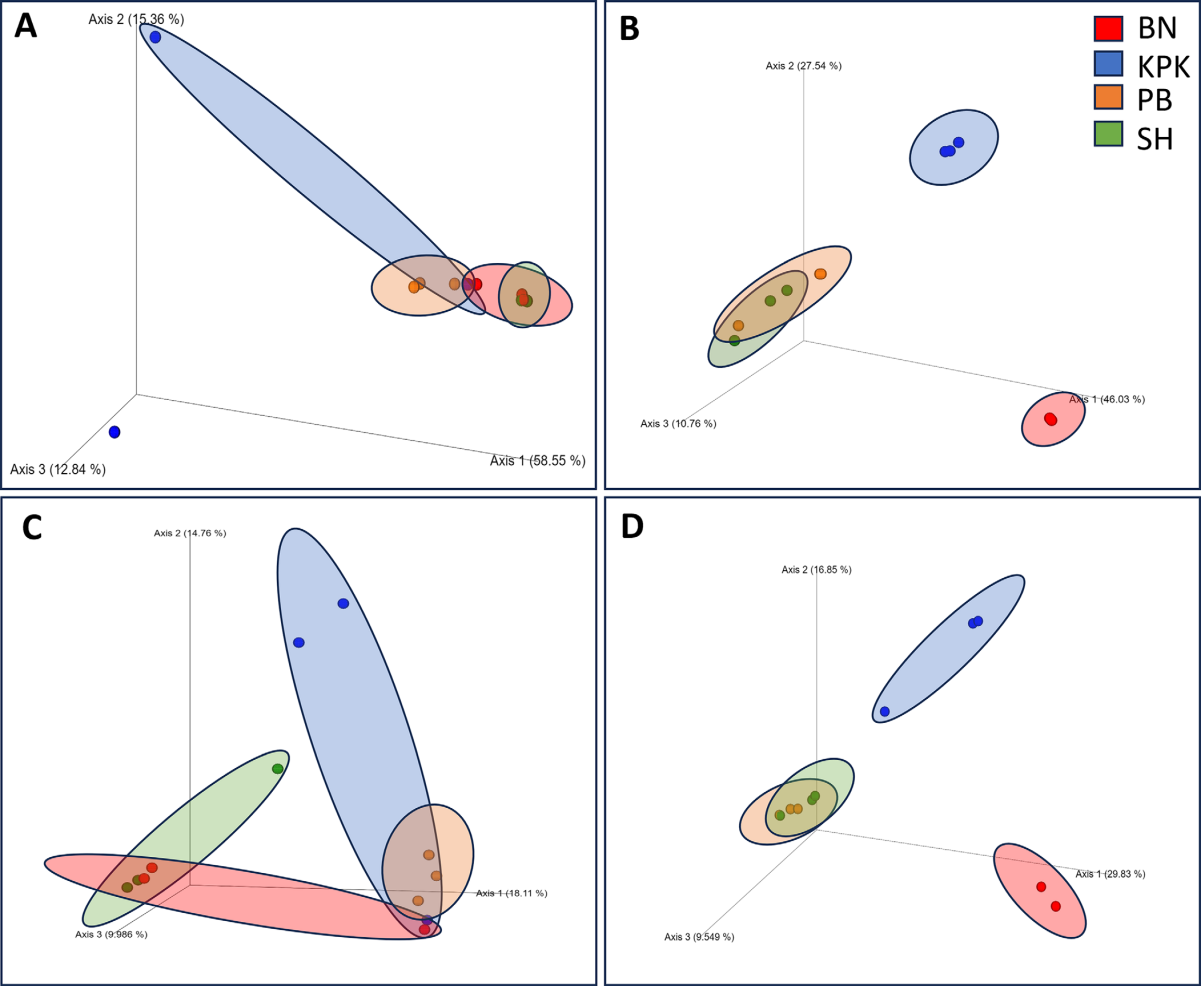
Principal coordinates analysis (PCoA) plots, A; *Rhipicephalus microplus* showed significant diversity on Bray–Curtis dissimilarity (*p*-value = 0.005) PERMANOVA test; otherwise most of the samples lie on the same axis and opposed dissimilarity. B; *Hyalomma anatolicum* Bray–Curtis clearly indicate that KPK and BN have higher dissimilarity as compared to PB and SH. C; *Rhipicephalus microplus* Jaccard distance indicates differences in microbial population based on presence and absence of ASVs. D; *Hyalomma anatolicum* Jaccard Distance clearly separated KPK and BN microbial populations.

The Bray–Curtis dissimilarity (*p*-value = 0.005) (Fig. 5A) and Jaccard distance (*p*-value = 0.024) matrices revealed significant differences in *R. microplus* microbial diversity and distribution across provinces and represented in PCoA plots (Fig. 5C). Differences were noted for Weighted UniFrac (*p*-value = 0.003) and Unweighted UniFrac (*p*-value = 0.04) (data not shown). Additionally, *H. anatolicum* ticks presented a significant Bray–Curtis (*p*-value = 0.001) (Fig. 5B) and Jaccard distance (*p*-value = 0.001) (Fig. 5D) in microbial communities across the provinces, 5D). Weighted and Unweighted UniFrac both resulted in significance with *p*-value = 0.003 (data not shown).

## Discussion

In our study, we analyzed *H. anatolicum* and *R. microplus* as these are two main ticks that infest the bovine population in Pakistan. The *H. anatolicum* tick*,* despite being a vector of lethal diseases like CCHF in humans [29] and theileriosis in cattle, is relatively understudied in terms of transcriptomic, genomic and microbiomic exploration. On the other hand, *R. microplus* has been widely studied for its microbiome, proteomic profile and genome database [25, 48, 51]. However, we aimed to investigate whether there are any differences in the microbiome profiles of *H. anatolicum* and *R. microplus* ticks in different regions of Pakistan*.*

Our findings indicate substantial variation in the microbiome, particularly in the case of *H. anatolicum*, suggesting that *Hyalomma* ticks in KPK harbor distinct bacterial communities compared to those previously reported. The beta diversity PCoA plots showed clear demarcation of KPK and Balochistan on an opposing axis. Balochistan has FLE as the top contributor to microbial counts (68%) and only 11.9% *Candidatus* Midichloria, whereas previous studies showed that their abundance was only higher if the tick is infected with *Theileria* protozoa [2]. *Hyalomma anatolicum* ticks of KPK presented *Acinetobacter* (16.3%) as the top contributor, unlike *Candidatus* Midichloria (0.43%) or *FLE* (1.16%), which contradicts the findings of previous studies about microbial communities of *Hyalomma* ticks [2, 9, 12]. In this study, only Punjab and Sindh *H. anatolicum* ticks showed somewhat conventional proportions of tick microbiome, consisting of a majority of Protobacteria (Rickettsiales, Francisellaceae and Moraxellaceae, in that order) for more than 80% in either case (Fig. 2). This scenario is better presented in the Bray–Curtis Matrix PCoA plot since Bray–Curtis also accounts for dissimilarity in species composition (Fig. 5B). The KPK and Balochistan microbial communities of *H. anatolicum* clustered on opposing coordinates. Punjab and Sindh, having predictable microbial communities, were cornered together on an axis away from KPK and Balochistan. It has already been well established that the tick microbiome is crucial for its health, reproduction and survivability [33, 34, 39, 52], and we suspect that this microbial change in Balochistan ticks has vitalized them more. The deviation from previous findings highlights the complex and dynamic nature of tick microbiomes, suggesting that environmental and geographic factors play a significant role in shaping the bacterial communities within *H. anatolicum* ticks. This variation may also reflect the ecological plasticity of this tick species, potentially enabling them to adapt to different habitats and climatic conditions. However, a comprehensive and controlled study is required to support this idea.

PCR amplification bias can also play a role in the distorted microbiome picture, but looking at other samples and absence of outliers in *H. anatolicum* removed this suspicion. Adding to that, there is a rich number of ASVs observed in this study from both ticks, especially when compared to previous studies [2, 42, 46]. However, a more accurate approach would be the use of Shotgun Metagenomic Sequencing to much more accurately analyze these microbial communities and abundance [15, 27]. The ticks from KPK showed a markedly more evenly distributed microbial population, where the top contributor was not only unpredictable, but had unexpectedly only 16% contribution, which is why KPK has the highest Shannon index points (Fig. 3), since the Shannon Index accounts for species richness in relation to their abundance (evenness) within the same sample. Usually, *H. anatolicum* ticks harbor one genus covering more than 50–70% of the total microbial population [5, 38], but surprisingly in this case of KPK ticks, not a single genus was able to occupy more than 16% of the total bacterial composition.

Another reason why Punjab and Sindh have similar microbial composition and abundance could be that these two provinces have regular cattle trade to and from one another. Movement of young calves, and beef and dairy cattle have for years hindered the development of a unique microbial population in these ticks. Balochistan in particular, has no evident trade of animals with any other province and with its harsh terrain and environment may have caused the ticks to harbor a slightly different microbiome over time. Our study is understandably insufficient to establish these patterns, but it presents the contrasting and interesting picture of *H. anatolicum* microbial composition.

On the other hand, our results for *R. microplus* with higher occurrence of *Coxiella-*like endosymbionts were in line with expected microbial communities reported previously [32]. However, two out of three pools of KPK province ticks contrasted, with *A. indicus* being the dominant species resulting in a skewed axis of KPK pools/samples on the Bray–Curtis Matrix PCoA plot (Fig. 5A). In most studies, both *Coxiella* and *Acinetobacter* occupy most (about 70–90%) of the *R. microplus* microbiome [42, 51]. Overall, the *R. microplus* across all provinces shared similar microbial composition; however, slightly changed abundance was observed (Figs. 1, 5A, 5C). This consistency across geographically diverse regions suggests that *R. microplus* ticks may maintain a more stable microbial composition regardless of environmental or ecological variations. Compared to *H. anatolicum*, *R. microplus* ticks may feed on a narrower range of hosts, reducing the diversity of microbial taxa they encounter. A stable host range could contribute to the consistency of their microbial communities across regions.

Given the role of tick microbiomes in vector competence, our findings raise important questions regarding the implications of these microbial differences for pathogen transmission dynamics in KPK and other regions of Pakistan. The variability in the *H. anatolicum* microbiome raises the possibility that regional differences in microbial communities could influence the tick’s ability to propagate more effectively and hence increase transmission rates of tick-borne diseases. The current study serves as a pilot study to further the investigation of *H. anatolicum* dynamic microbial adaptation, their effect on vectorial potential and overall survivability of ticks, with a larger sample size.

## Conclusion

This study provides new insights into the geographic variation of tick microbiomes in Pakistan. While *H. anatolicum* ticks from the western provinces (Balochistan and Khyber Pakhtunkhwa) exhibit substantial microbial diversity, possibly influenced by environmental and ecological factors, *R. microplus* ticks maintain a more stable microbial community regardless of geographic location. This significant change, in a context where these western provinces have been reporting increases in cases of CCHF and theileriosis, may play a role in this adaptability and vectorial potential. These findings suggest that tick microbiomes are shaped by a combination of species-specific factors and environmental pressures, with important implications for the transmission of tick-borne diseases. Future research should focus on understanding the functional roles of microbial taxa in shaping tick vector competence and the potential for leveraging microbial interventions in tick control strategies.
